# Early mobilization post-myocardial infarction: A scoping review

**DOI:** 10.1371/journal.pone.0237866

**Published:** 2020-08-17

**Authors:** Haroon Munir, Jake Fromowitz, Michael Goldfarb

**Affiliations:** 1 Division of Experimental Medicine, McGill University, Montreal, QC, Canada; 2 Nova Southeastern University, Fort Lauderdale, FL, United States of America; 3 Division of Cardiology, Jewish General Hospital, McGill University, Montreal, QC, Canada; Hospital Clinico San Carlos, SPAIN

## Abstract

Bedrest and immobilization following a myocardial infarction (MI) can lead to functional impairment that can persist following hospitalization. Early mobilization (EM) is associated with good functional and clinical outcomes in critical care, medical and surgical settings. However, the impact and current role of EM in post-MI care has not been well-defined. Our objective was to assess the evidence for post-MI mobilization, define current post-MI mobilization practice, and understand perspectives of cardiovascular professionals toward mobilization. A scoping review related to “early mobilization” and “myocardial infarction” was performed using the Joanna Briggs Institute Methodology. Pubmed, Embase, Google Scholar, Cochrane Library and CINAHL databases were included. Results were categorized into six topic areas. There were 59 references included in the analysis. There was evidence for the effectiveness and safety of earlier mobilization in experimental studies of the pre-revascularization era, but there was a lack of strong evidence for EM in contemporary post-MI care. Mobilization appears to be safe following arterial catheterization and is associated with minimal hemodynamic and respiratory compromise. Most people are delayed in mobilizing post-MI and spend the majority of the initial hospitalization period lying in bed. Only 1 of 7 current major cardiovascular professional societies guidelines recommend EM post-MI. There were no studies exploring the perspectives of cardiovascular professionals toward mobilization. EM may be beneficial in the post-MI care. However, there is an evidence gap for the impact of EM post-MI in the contemporary literature. More robust evidence from randomized clinical trials is required to inform clinicians and influence practice.

## Introduction

“The bed is not a resting place for the patient with cardiac disease” Drs. Levine and Lown (1952) [1].

Bedrest and immobility has been part of the culture of care following myocardial infarction (MI) for the past century [2]. Mobilization too soon following an MI was traditionally considered dangerous due to the risk of coronary ischemia, arrhythmia, and aneurysm formation [2]. Yet despite procedural and therapeutic advances that have decreased length of hospital stay and improved clinical outcomes, involuntary bedrest and delayed mobilization continue to be part of acute cardiology care culture [3, 4].

Bedrest and immobilization, in combination with acute illness, lead to muscle catabolism within hours of hospital admission, which results in rapid loss of skeletal muscle mass and reduced strength [5]. Older adults are particularly susceptible to muscle loss and are at increased risk of disability at hospital discharge [6]. This functional impairment can persist for years, impairing quality of life and reducing functional independence [7].

Early mobilization (EM) is a care process that involves initiation of mobilization activities as soon as hemodynamic and respiratory stabilization is achieved, typically with 1–2 days of admission [8]. The goal of EM is to prevent loss of muscle strength and prehospital mobility capabilities and to improve post-hospital functional status. In acute and intensive care settings, there is evidence that EM improves muscle strength and physical function, reduces rates of delirium, and decreases hospital length of stay and readmission rate [9–11]. The safety and feasibility of EM in critically ill patients has been established [12]. As a result of this evidence, critical care professional societies recommend EM as part of standard practice in intensive care units [13, 14]. In the cardiovascular intensive care unit, major cardiovascular (CV) professional society guidelines do not provide recommendations for mobilization in hospital following an MI [15–17]. One exception is the European Society of Cardiology guidelines, which recommends EM for most post-ST segment elevation MI patients, but does not cite any evidence to support this recommendation [18].

To better understand the potential role and benefits of mobilization post-MI, we performed a scoping review of the literature to (1) assess the evidence for post-MI mobilization, (2) define current post-MI mobilization practice, and (3) understand current beliefs, attitudes, and knowledge of CV professionals toward mobilization. Knowledge gaps in our understanding of post-MI mobilization are presented to inform future research directions.

## Methods

### Search strategy

A comprehensive search strategy was devised in consultation with a medical research librarian and established a priori to ensure maximum sensitivity (S1 Fig). We assessed papers containing the terms “early mobilization” and “myocardial infarction” either in the title, abstract or body of the paper. We also conducted searches that included “mobility OR mobilization” with “intensive care unit OR ICU.” Selection of papers were based upon the population, concept and context guidelines specified in the Joanna Briggs Institute Methodology for JBI Scoping Reviews [19]. Papers selected included human patients without any age restriction, undergoing post-MI mobilization interventions with outcomes assessing the efficacy of these interventions. There were no geographic, gender, cultural, ethnic or specific language restrictions, however, only non-English studies from the contemporary period (year 2000 and beyond) were included in the analysis.

### Information sources

Sources of information included but were not limited to primary research studies, clinical trials, systematic reviews, case-studies, meta-analysis. Information sources were intentionally left open to prevent the possible omission of relevant records.

### Databases

We consulted Ovid MEDLINE (Embase Classic + Embase (1947 to April 2019), Ovid Healthstar (1966 to May 2019) and Ovid MEDLINE (1946 to 2019), PubMed, Google Scholar, Cochrane Library and CINAHL databases. The selected search strategies for Ovid MEDLINE and CINAHL are outlined in S1 Fig.

### Search and selection of sources of evidence

Our primary search consisted of records related to “Early mobilization and myocardial infarction.” Additional searches were conducted on EM in the intensive care unit, mobilization with cardiac devices relevant to MI, and hemodynamic studies on EM, including those after MI. We compiled all the records we obtained from Ovid MEDLINE, CINAHL, Google Scholar and PubMed databases into EndNote X9. We began deduplication in EndnoteX9, exported the results into Microsoft Excel and selected relevant sources based upon the topic of interest of the review, narrowing it down to 343 records. Non-relevant references were excluded. Studies were reviewed by two independent reviewers (H.M. and J.F) for inclusion criteria. Disagreements were resolved by a third reviewer (M.G.). Bibliographies of included studies were manually searched, and relevant studies were reviewed for inclusion.

### Data charting process and synthesis of results

We categorized the references into 6 topic areas: (1) Historical Evidence and Recommendations for EM Post-MI, (2) Modern EM Practices Post-Myocardial Infarction, (3) Hemodynamic Impact of EM Interventions, (4) Mobilization Practices with Cardiac Devices, (5) Professional CV Society Guidelines for EM, and (6) Current Beliefs, Attitudes, and Knowledge of CV Professionals Toward Mobilization. We defined contemporary literature pertaining to EM practices as papers dated after 2000, given the emergence of percutaneous coronary intervention procedure in the 1990s.

## Results

**Fig 1:**
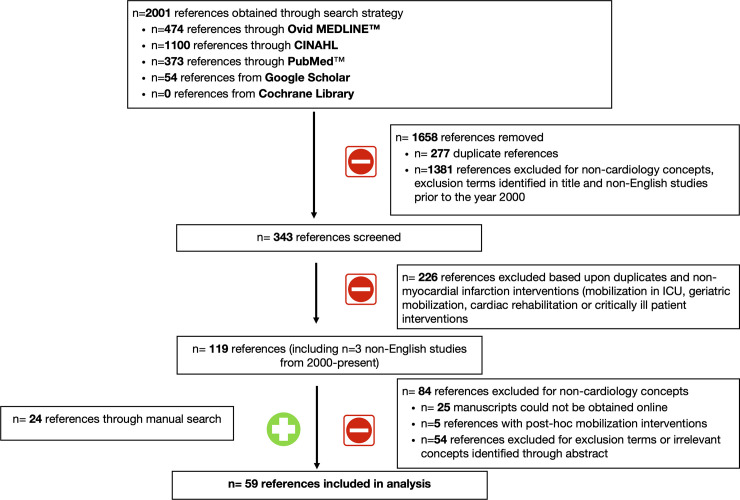
Search strategy flowchart.

There were 59 references included in our analysis (35 references related to our search strategy; 24 references via manual search; Fig 1).

### Historical evidence and recommendations for early mobilization post-myocardial infarction

In 1929, four to six weeks of bedrest was recommended for the management of acute coronary thrombosis (S1 Table) [20]. In the 1960s, Brummer et al. reported that mobilizing people post-MI at day 12 compared to day 16 was safe [21]. Irwin et al. postulated that routine prolonged bedrest post-MI may be unnecessary and potentially harmful to patients’ mental and physical well-being [22]. Levine et al. suggested that sitting in an armchair post-MI could result in improved cardiac recovery compared to lying in a bed [1]. In the 1970s, several RCTs were performed evaluating earlier (7 to 10 days) vs. later (13 to 20 days) post-MI mobilization. These studies found no difference morbidity, mortality, and risk of complications [23–26]. There were anecdotal reported that earlier mobilization out of bed post-MI resulted in improvements in patient’s functional status and psychological benefit. However, there were no objective patient-centered outcomes reported in these studies [26].

### Contemporary evidence and practice of early mobilization post-myocardial infarction

Published studies on post-MI mobilization strategies in the past three decades have been mainly systematic reviews of earlier studies; there was one RCT (Table 1). A 2003 systematic review and a 2009 Cochrane review looked at outcomes of post-MI patients undergoing shorter (2 to 7 days) vs. longer periods (8 to 12 days) of bedrest [27, 28]. These reviews found no evidence that shorter bedrest was more harmful than longer bedrest in terms of mortality, reinfarction, thromboembolic events or mortality. A systematic review with meta-analysis by Cortes et al. found 14 experimental studies of mobilization strategies post-MI and found a trend toward decreased mortality in the earlier mobilization group [29]. However, studies included in these reviews were mainly conducted prior to the coronary revascularization era.

**Table 1:** Modern early mobilization practices post-myocardial infarction.

Study / Year	Study Type	No. & Population	EM Intervention	EM Results/*Recommendation*
Herkner, H. **2003**	Systematic review & meta-analysis	2658 pts. with uncomplicated MI	Short period of bedrest (2–12 days) or prolonged bedrest (5–28 days).	No evidence that shorter bedrest was more harmful than longer bedrest in terms of mortality, reinfarction, post-infarction angina or thromboembolic events.
Herkner, H. **2007**	Cochrane Review	2958 pts. post-acute uncomplicated-MI	Short period of bedrest (median 6 days) or prolonged bedrest (median 13 days).	No evidence that shorter bedrest was more harmful than longer bedrest in terms of all-cause mortality, cardiac mortality or reinfarction.
Lopes, JL. **2008**	Literature Review	2233 pts. with AMI	2–10 days of bedrest in EM group; 5–28 days bedrest in long-resting group.	No evidence of complications related to short periods of bedrest in patients with acute MI.
Cortes, OL. **2009**	Systematic review & meta-analysis	3148. pts. following AMI from 14 studies	Varied depending upon study.	Trend towards decreased mortality with EM after AMI.
Asgari, M **2014**	Randomized clinical trial	38 pts. with AMI admitted to CCU	Pts. randomized to intervention group (mobilize 12–18 hours post-CCU admission) or routine care (48 hours post-CCU admission)	EM was effective in reducing depression in patients and recommended its use in the care of patients with AMI.
Cortes, OL. **2015**	Observational pilot study	31 diagnosed AMI pts. admitted to CCU	Bedrest, semi-fowler, transfer to chair, and standing/walking.	Patients experiencing uncomplicated AMI spend majority of 72 hour stay in the CCU in bed.

ACT, Acute coronary thrombosis; AMI, Acute myocardial infarction; CCU, Cardiovascular Care Unit; EM, Early Mobilization; MI, Myocardial Infarction; Pts, Patients.

To characterize current post-MI mobility practice, Cortes et al. conducted a pilot study of 31 acute MI patients in three academic cardiac care units in Canada [30]. They reported that the first attempt at mobilization occurred on average 50 hours post-symptom onset and 21 hours post-admission to the cardiac care unit. People with uncomplicated acute MIs spent nearly 70% of their time during the first 72-hours of admission in bed. Asgari et al randomized patients in a coronary care unit to receive either an EM intervention or usual bed rest care and found less depressive symptoms in the earlier mobilized group [31].

### Hemodynamic impact of early mobilization interventions

There were 5 studies evaluating the hemodynamic impact of EM; only one of the studies specifically focused on post-MI patients (Table 2). A prospective study of 31 intensive care patients who were deemed to have limited cardiac and respiratory reserve found heart rate and blood pressure increased and oxygen saturation was lower during mobilization, although changes were not considered significant [32]. A retrospective study of 31 critically obese patients showed that there were significant increases in respiratory rate, oxygen saturation, and respiratory reserve following mobilization as compared to initial values [33]. An observational study of 53 post-elective cardiac surgery patients undergoing an EM intervention consisting of early post-op chair sitting found reductions in right atrial pressure, but a decrease in central venous oxygen saturation and an increase in arterial lactate [34]. A retrospective study in Japan evaluated the physiological changes during EM sessions in mechanically ventilated patients and found no significant changes in heart rate or blood pressure, although there were improvements in oxygenation parameters [35].

**Table 2:** Hemodynamic impact of early-mobilization interventions.

Study/Year	Study Type	No. & Population	Place of Admission	EM Intervention	Hemodynamic Impact
Stiller, K. **2004**	Prospective study	31 intensive care patients.	Intensive care unit	Sitting on the edge of the bed and standing	Significant increases in heart rate, blood pressure. Decreases in percutaneous oxygen saturation in early mobilization patients.
Genc, A. **2012**	Retrospective study	31 critically obese patients.	Intensive care unit	37 mobilization sessions in their physiotherapy program during intensive care unit stay.	Significant increase of SpO_2_, respiratory rate and respiratory reserve in patients receiving mobilization sessions compared to initial values.
Cassina, T. **2016**	Observational study	53 patients after elective cardiac surgery.	Cardiovascular intensive care unit	Patients placed sitting on the bed for 5 min, moved to an armchair for 30 min, and finally returned to the initial recumbent position on 1^st^ post-operative day	Significant increases in arterial lactate along with reduction in right atrial pressure and ScvO_2_; HR and SpO_2_ unchanged in mobilization group.
Umei, N **2016**	Retrospective study	23 patients requiring mechanical ventilation.	Intensive care unit	Progression from seated on edge of hospital bed, transfer to chair, then to ambulation.	No significant changes in heart rate, arterial blood pressure. Increase partial pressure ratio of arterial blood/inspired fraction of oxygen ratio—indicated improved lung function.

### Mobilization practices with cardiac devices relevant to post-myocardial infarction care

Studies reporting mobilization strategies following femoral and radial cardiac catherization were mainly performed in elective coronary angiography or percutaneous intervention (Table 3). In patients undergoing elective percutaneous transluminal coronary angioplasty, mobilization as soon as 6 hours after sheath removal was found to be safe and feasible [36]. Earlier mobilization post-7 French catheterization and percutaneous transluminal coronary angioplasty increased patient comfort and significantly reduced pain [36, 37]. Earlier mobilization following percutaneous coronary intervention had no effect on the incidence of either hematoma formation nor bleeding at the puncture site [38]. In the early 2000s the introduction of radial catherization for coronary angiography increased the potential to mobilize patients earlier post-procedure [39]. In a group of older adults post-MI, Kagoshima *et al*. compared a multidimensional protocol including a transradial approach and earlier mobilization with a transfemoral approach, bedrest and late mobilization, and found that the earlier mobilization group had shorter lengths of intensive care unit and hospital stay and lower rates of systemic complications, including delirium [40]. Mobilization in people with femoral central venous catheters in acute care settings was also shown to be safe [41, 42].

**Table 3:** Mobilization practices with cardiac devices.

Study/Year	Cardiac Device	No. & Population	EM Intervention	EM Results/*Recommendation*
Perme. **2013**	Femoral venous catheter	77 pts. with femoral catheters in the cardiac intensive care unit	210 physiotherapy activities with 630 mobility activities (sitting at bed side, standing at bedside, transfer to chair, walking).	No catheter related adverse events. Early mobilization after femoral catheter intervention is important in minimizing functional decline
Damluji, A. **2013**	Femoral venous catheter	101 pts. with femoral catheters in the medical intensive care unit	In-bed exercises, supine cycle ergometry, sitting and standing/walking.	No catheter-related adverse events.
Fowlow, B. **1995**	Femoral arterial catheter	85 pts. admitted to intensive care unit after elective percutaneous transluminal coronary angioplasty (PTCA)	Randomly assigned pts. to 6 or 8 hours after sheath removal	Ambulation 6 hours post-sheath removal resulted in no significant increases in hematoma formation at puncture site compared to group ambulated 8 hours post procedure. Early mobilization group had significantly lower pain scores than late group at 8 hours.
Mah, J. **1999**	7 French (F) arterial catheter	880 patients post-7F catheter procedure	3-hour ambulation post procedure (early) or 5-hour ambulation (late)	Early mobilization group had significantly lower bleeding and hematoma formation compared to late mobilization group. Concluded that early mobilization post-cardiac catherization is safe, can decrease hospital stay and increase patient comfort.
Kagoshima, M. **2000**	Radial artery catheters Femoral arterial catheter	89 patients, 32 of which treated with new transradial approach, 57 treated by old protocol	Rapid mobilization and discharge involve walking on ward on third day following procedure & encouragement of discharge within 2 weeks.	Shortened hospital stay with no increase in in hospital mortality, cardiac events or decline of left ventricular function.
Kim, K. **2013**	Various catheters and sheaths	Variable (metanalysis)	Variable bed rest durations and early mobilization protocols	Early mobilization following percutaneous coronary intervention had no effect on hematoma formation or bleeding at puncture site.

AMI, Acute myocardial infarction; BP, Blood pressure; pts, Patients.

### Professional cardiovascular society guidelines for early mobilization post-myocardial infarction

Only 1 out of 7 current CV professional society guidelines for acute MI has recommendations for EM post-MI (Table 4). The 2017 European Society of Cardiology ST elevation MI guidelines recommend mobilization of patients 1 day after an acute MI in the “majority of patients”[18]. These guideless allow that prolonged bedrest may be needed with patients with severe infarcts or major complications. No evidence is cited to support these recommendations.

**Table 4:** Professional cardiovascular society guidelines for early mobilization.

Professional Society, Author	Date of Recommendation	EM Guidelines, Recommendation
American College of Cardiology, STEMI Guidelines, O’Gara [43]	**2013**	No mention of mobilization or ambulation in the management of post-STEMI patients
American College of Cardiology, NSTEMI Guidelines, Amsterdam [17]	**2014**	No mention of mobilization or ambulation in the management of post-STEMI patients
European Society of Cardiology, NSTEMI Guidelines, Roffi [16]	**2015**	No mention of mobilization or ambulation in the management of post-STEMI patients
American College of Cardiology, American Heart Association. Society for Cardiovascular Angiography and Interventions, Levine et. Al [44]	**2015**	No mention of mobilization or ambulation in the management of post-STEMI patients
National Heart Foundation of Australia and Cardiac Society of Australia and New Zealand: Australian clinical guidelines for the management of acute coronary syndromes—Chew 2016 [45]	**2016**	No mention of mobilization or ambulation in the management of acute coronary syndromes
European Society of Cardiology, STEMI Guidelines, Ibanez [18]	**2017**	Early ambulation (day 1) recommended in majority of patients. Bed rest recommended in patients with extensive myocardial damage, heart failure, hypotension, or arrhythmias.
		No evidence given to specifically support these recommendations, however cardiac rehabilitation after STEMI is a Class I, Level A recommendation.
Canadian Cardiovascular Society, STEMI Guidelines, Wong [15]	**2019**	No mention of mobilization or ambulation in the management of post-STEMI patients

NSTEMI, Non-ST-elevation myocardial infarction; STEMI, ST-Elevation Myocardial Infarction.

### Current beliefs, attitudes, and knowledge of CV professionals toward mobilization

There were no studies that specifically focused on the beliefs, attitudes, or knowledge of CV providers towards mobilization.

## Discussion

The aim of our study was to assess the evidence for EM post-MI, understand current post-MI mobility practice, and determine perspectives of CV healthcare professionals towards mobilization. We found that the majority of post-MI mobilization studies were from the pre-coronary revascularization era and there were few contemporary studies evaluating the role of post-MI mobilization. Many of the older studies were experimental, whereas recent studies were observational. Current CV professional society guidelines largely do not provide recommendations for post-MI mobilization. Evidence for current mobility practice was limited but suggested that bedrest and delayed mobilization is still common in post-MI care. There are a lack of studies exploring the perspectives of CV healthcare professionals towards mobilization.

In the early post-MI period, there is evidence that patients are not being mobilized. Cortes et al. looked at the time to first ambulation post-MI in three Canadian academic tertiary care centers [3]. Only one-quarter of patients walked during the first 48 hours of hospitalization and the majority of post-MI patients (>50%) did not ambulate by 4 days post-MI. Patients who were less likely to ambulate were older and had arrhythmias or were receiving inotropic drugs. The majority of both daytime and nighttime periods were spent in bed (61.5% morning, 64.5% afternoon, 79.9% night). Nearly half of post-MI patients received a prescription for involuntary bedrest. Despite a wide search strategy, there were no other published studies exploring post-MI mobility practices. Whether this single study’s findings are indicative of post-MI mobility care in other healthcare settings is uncertain.

Studies from the pre-coronary revascularization era showed the safety, feasibility and benefits of earlier mobilization post-MI. These studies found that earlier post-MI mobilization resulted in reduced length of hospital stay without an increase in in-hospital complications or short-term post-discharge complications [21, 46, 47]. However, these studies were performed when intensive care and hospital length of stay was considerably longer than in contemporary care. The length of hospital stay post-MI has decreased substantially in the United States with current median post-MI stay for all-comers at 3 days (interquartile range 2 to 6) [48]. Within this short timeframe, it is possible that earlier mobilization may not make a considerable difference in outcomes. However, older adults have a median duration of length of stay post-MI of 6 days and are more likely to have prolonged length of hospital stay (> 7 days) [49]. People with pre-hospital functional impairments have even longer length of hospital stays [50]. In contemporary datasets from other healthcare settings, median length of hospital stay post-MI can be as long as 13 days [51, 52]. Thus, there may be an opportunity for earlier mobilization to decrease length of hospital stay in certain populations.

Beyond resource utilization, EM has been shown in other clinical settings like the intensive care unit and the general medical ward to minimize functional decline, improve psychological wellbeing, prevent post-hospitalization syndrome, and decrease hospital readmission [9, 11, 53]. EM may also achieve these patient-important outcomes post-MI. However, our review highlighted the lack of high-quality studies exploring the timing and potential benefits of EM post-MI in the modern era. There is some observational evidence for EM in acute cardiac populations. A retrospective study of 264 older adults (mean age 77; 19% post-MI patients) undergoing EM in a quaternary care American cardiac intensive care unit found that more than 40% of patients had improvements in functional status during unit stay [54]. The majority of patients had regained more than three-quarters of the prehospital functional level by the time of unit discharge. Frail older adults, who had lower functional abilities at baseline compared to their non-frail counterparts, had similar overall improvements in functional status. Importantly, there were no patient falls, dislodgement of lines, drains, or endotracheal tubes, or injuries to healthcare personnel related to EM activities in this acute cardiac population.

Our review found that EM resulted in small alterations in heart rate, blood pressure and oxygen saturation, but these changes did not seem to be of major clinical importance [32, 55]. These hemodynamic results serve to further support EM’s safety in acute cardiac care. Mobilization with devices that may be relevant to post-MI care in complex patients, such as those receiving percutaneous mechanical ventilation, mechanical circulatory support and continuous renal replacement therapy, have also been shown to be safe [12, 56, 57]. Even mobilization in people receiving vasoactive medications is not associated with hemodynamic instability [58]. However, additional data are needed in people with ischemic heart disease, especially people who were not fully revascularized and may be at increased risk of active ischemia and arrhythmia. We also did not identify any recent studies investigating early mobilization following MI or percutaneous intervention by radial access. Understanding the potential role and safety of EM following radial access for MI has potential clinical practice implications. There is a need for RCTs to address these issues and explore the safety and benefits of EM in post-MI patients in contemporary care. These studies should investigate whether specific patient populations, such as older adults, frail patients, and people with pre-existing functional limitations may benefit from earlier attempts to mobilize.

Current mobilization practices following MI are unknown. With current radial access techniques, it may be safe to transfer the patient post-percutaneous intervention directly from the cardiac catheterization lab to a sitting position in an armchair or in bed. The current practice in our institution is to permit an uncomplicated MI patient who underwent radial arterial catheterization followed by use of a radial artery occlusive device to mobilize to the chair within 1 hour of procedure. Radial artery hemostasis clamp duration of 60 minutes is associated with a low rate of radial artery occlusion and could promote earlier post-MI mobilization [59].

Despite the weight of historical clinical evidence, the lack of a strong evidence base for post-MI mobilization may explain why there is a lack of CV professional society recommendations for mobilization. Of the 7 current CV professional society MI guidelines, only one, the European Society of Cardiology, had a recommendation for EM. However, this recommendation was not accompanied by supporting evidence. Interestingly, an older version (2004) of the American College of Cardiology ST-elevation MI guideline recommends that patients free of ischemic discomfort, symptoms of heart failure or serious arrhythmia should not exceed 12 to 24 hours of bedrest [60]. Stronger evidence for EM’s effectiveness post-MI are likely needed to influence CV professional society guideline recommendations.

There were no studies identified that examined the beliefs, attitudes, and knowledge of CV providers towards mobilization. Barriers to mobilize have been identified in critical care providers that may be relevant to acute CV practice. Half of critical care providers do not perceive EM of patients as a top care priority [61]. Three-quarters of critical care physicians report that they lack adequate knowledge or training in mobilizing patients [62]. Common perceived barriers to EM amongst critical care physicians were safety concerns (hemodynamic instability, line dislodgements), medical instability, and limited staffing, and insufficient guidelines to support mobilization [63, 64]. The most commonly cited barriers for implementation and performance of EM amongst critical care nurses were high workload, patients' inability to exercise, lack of time, inadequate nurse to patient ratio, and absence of relevant education [65]. In acute care cardiology, there is a need to understand and address structural, provider, and patient-level barriers to mobilization.

Knowledge gaps of the role of EM post-MI exist and should be addressed in future studies. Specific subgroups may stand to benefit more from earlier attempts to mobilize. These include older adults, particularly those with frailty, people with limitations in prehospital functional ability, and those with a longer predicted hospital length of stay. Further research is required to operationalize EM, as no consensus for a standardized definition exists in the literature. There is also a need to ascertain whether EM can improve patient-centered outcomes, such as post-hospital functional status and quality of life. Older adults, in particular, prioritize individual quality of life and functional independence over other more conventional societal measures [66]. In addition, whether involving family members in the mobilization process improves outcomes should also be explored. A study in the critical care setting showed that 84% of family members wish to be engaged in care [67]. Recent critical care society guidelines also recommend engaging family members in care to improve patient and family member outcomes (i.e., mental health) [68]. Nurse-driven approaches to EM in post-MI care may also be considered as a pragmatic approach in less resource rich settings [69, 70].

Many institutions transfer patients to intermediate or step-down units following ICU stay, which can provide further opportunity for mobility progression. While EM is practiced in 20–50% of ICUs, the current prevalence of EM in cardiac ICUs or intermediate care units is unknown [71]. Further studies are needed to determine the optimal mobility trajectory following an MI.

There are limitations to our scoping review. First, despite our search strategy being designed for maximum sensitivity, one-third of the references were included from the manual search. Inclusion of these additional references were mutually agreed upon by two reviewers. Second, the strength of our conclusion was limited by the availability of studies in the published literature and thus were affected by the paucity of data in some sections. For example, only one study concerning current mobility practices post-MI was included in the analysis. There were 25 articles excluded because they were not published online. These articles were considered to be not relevant to the study based on examination of their title and abstract. There were no language restrictions, however, only non-English studies from the contemporary period (year 2000 and beyond) studies were included. Third, for some of the older studies, only the abstract and not the full manuscript was available for analysis.

## Conclusion

The main body of evidence for EM post-MI comes from the pre-revascularization era and supports the efficacy of earlier mobilization. However, there is an evidence gap for the feasibility, safety, and outcomes for EM post-MI in contemporary care. More robust evidence is required from RCTs about the role of EM post-MI, particularly in subgroups that may stand to benefit the most, in order to inform professional CV society recommendations and influence clinical practice.

## Supporting information

S1 Fig Selected search strategies & PRISMA checklist. (PDF)

S1 Table Historical evidence and recommendations for early mobilization post-MI. (PDF)
