# Supplementary material for: Early mobilization post-myocardial infarction: A scoping review
Source: PLoS One. 2020 Aug 17;15(8):e0237866. doi: 10.1371/journal.pone.0237866 (PMC7430744; doi:10.1371/journal.pone.0237866)
Supplement: S1 Fig — (PDF) [file pone.0237866.s001.pdf]

S1. Fig. Selected Search Strategies & PRISMA Checklist

<u>Ovid MEDLINE Search Strategy</u>

Database(s): **Embase Classic+Embase** 1947 to 2019 July 09**, Ovid Healthstar** 1966 to May 2019**, Ovid MEDLINE(R) ALL** 1946 to July 09, 2019

#	Searches	Results
1	(coronary adj3 syndrome*).tw,kf.	110423
2	myocardial infarction*.tw,kf.	585916
3	exp Myocardial Infarction/	708314
4	1 or 2 or 3	924230
5	((early or earlie$ or accelerat$ or immediat$ or fast track or timing or rapid$) adj5 (mobil$ or ambulat$ or rehab$ or physiotherapy or physical therapy or physical activity or movement or sitting or standing or walking or semi recumb$ or out of bed)).tw.	114220
6	[(bedrest/ or immobilization/ or rest/ or bedrest or bedrest or bed bound or bedbound).tw. and (time factors/ or time/ or early.tw,kf.)]	0
7	5 or 6	114220
8	4 and 7	2251
9	early mobilization/ or mobility/ or mobilization/	41464
10	8 and 9	391
11	Exercise/ or exercise/ or training.mp.	1676966
12	Rehabilitation/ or Rehab.mp. [mp=ti, ab, hw, tn, ot, dm, mf, dv, kw, fx, dq, nm, kf, ox, px, rx, ui, sy]	132114
13	11 or 12	1789248
14	7 or 13	1890744
15	4 and 14	23911
16	**9 and 15**	**474**

CINAHL Search Strategy:

”"(""(""exp Myocardial Infarction OR (coronary adj3 syndrome).tw,kf. OR myocardial infarction*.tw,kf. OR (bedrest/ or immobilization/ or rest/) OR ((bedrest or bedrest or bed bound or bedbound).tw.) OR (((early or earlie$ or accelerat$ or immediat$ or fast track or timing or rapid$) adj5 (mobil$ or ambulat$ or rehab$ or physiotherapy or physical therapy or physical activity or movement or sitting or standing or walking or semi recumb$ or out of bed)).tw.) OR (exp Intensive Care Units/ or Critical Illness/ or exp Critical Care/ or (critical* adj3 (ill* or care*)).tw. or intensive care.tw. or (icu or icuaw).tw) OR (Early mobility/ or early mobilization.mp. [mp=title, abstract, original title, name of substance word, subject heading word, floating sub-heading word, keyword heading word, organism supplementary concept word, protocol supplementary concept word, rare disease supplementary concept word, unique identifier, synonyms]) OR (Exercise/ or exercise/ or training.mp.) OR (Rehabilitation/ or Rehab.mp. [mp=title, abstract, original title, name of substance word, subject heading word, floating sub-heading word, keyword heading word, organism supplementary concept word, protocol supplementary concept word, rare disease supplementary concept word, unique identifier, synonyms]) OR (respiratory/ or respiration/) OR pulmonary"") AND (time factors/ or time/ or early.tw.)"") AND (exp Intensive Care Units/ or Critical Illness/ or exp Critical Care/ or (critical* adj3 (ill* or care*)).tw. or intensive care.tw. or (icu or icuaw).tw.)”*

Search and Selection of Sources of Evidence

We began with Ovid MEDLINE, using a search strategy consisting of terms search in either for keyword, abstract or in the title of the paper. Generating a search list of terms began after consulting the methodology section of two Cochrane Reviews[72, 73] regarding rehabilitation interventions after myocardial infarction. Nine versions of combining these search terms were constructed to reveal results most representative of the papers we sought to review.

We applied a similar search strategy of terms to CINAHL with a number of limiters. We began searching with records using the terms *“(myocardial infarction or heart attack or mi) AND (early mobilization or early ambulation or early mobility)”* with a number of limiters. The limiters to the search query included age limits (restricted to Adolescent: 13-18 years, Adult: 19-44 years, Middle Aged: 45-64 years, Aged: 65+ years, Aged, 80 and over), journal subjects (Allied Health, Alternative/Complementary Therapies, Asia, Australia & New Zealand, Biomedical, Blind Peer Reviewed, Canada, Continental Europe, Core Nursing, Double Blind Peer Reviewed, Editorial Board Reviewed, Europe, Expert Peer Reviewed, Health Promotion/Education, Health Services Administration, Nursing, Peer Reviewed, Public Health, USA), clinical subjects (Human) and language (English).

With our search strategy on CINAHL, we generated 1110 records in total, and screened these records in Endnote X9, eliminating records with irrelevant terms or topics. Using the search function in Endnote X9,

we removed records with the following terms: *stroke, neuro, diabetes, achilles, spinal, amputation, knee, multiple sclerosis, heel cancer, amputee, hip, ankle, limb, bone, fractures, laparo, fall, cerebral, rheu, polio, delirium, HIV, syndrome, chondrocyte, Alzheimer, child, visual, ligament, postpartum, onco, tendon, parkinson, infants, muscular dystrophy, arachnoid, bowel, pediatric.* This narrowed the total number of records down to 428.

We then consulted Google Scholar using the search term *early mobilization after myocardial infarction* and screened the first 100 records for relevancy and included 13 records in our review. Three additional separate searches were conducted on Google Scholar using different search terms: 1) *early mobilization/ or mobility/ or mobilization) AND (intensive care unit/ or ICU),* 2) *(myocardial infarction/ or heart failure) AND (mobilizing/ or mobility)* and 3) *Intensive Care Unit Outcomes*. We obtained 19, 900 search results for 1) and went through the first 50 pages and selected 39 records in total that were relevant. For 2), we obtained 18, 700 results and went through the first 20 pages to obtain 1 relevant result. Lastly, for 3) we obtained 2, 410, 000 results and went through the first 15 pages to obtain 1 relevant result.

We ran a search through Cochrane Library using the search term *early mobilization AND myocardial infarction* and obtained 0 Cochrane reviews.

Lastly, we consulted PubMed® using the search term "*myocardial infarction"[MeSH Terms] OR ("myocardial"[All Fields] AND "infarction"[All Fields]) OR "myocardial infarction"[All Fields] and early mobilization* and generated 373 records.

Combining all records from into EndNote, we categorized references based upon certain limiters in order to exclude irrelevant or out-of-scope records, as well as references not in English or French:

Exclusion Category	Terms
Language	Polish, German, Italian, Persian, Hebrew, Spanish, Russian, Japaneese, Norwegian, Swedish, Korean, Czech
Terms (excluded records containing these terms in title)	Stroke, neuro, diabetes, achilles, spinal, amputation, knee, multiple sclerosis, heel cancer, amputee, hip, ankle, limb, bone, fractures, laparo, fall, cerebral, rheu, polio, delirium, HIV, chondrocyte, Alzheimer, child, visual, ligament, postpartum, onco, tendon, Parkinson, infants, muscular dystrophy, arachnoid, bowel, pediatric

Non-Cardiology Concepts/Out of Scope	Cellular cardiology (endothelial cell progenitors), cellular biology cholecystitis, gait training, paraplegic, neurological disorders, grafting pain management, music, anesthesia, stroke, pharmacology, pain management, cardiac catheterization, cardiac boomers, gastrointestinal disorders, tuberculosis, technological mobility devices, orthopaedics, prosthesis, injuries and trauma, stem cell mobilization, pneumonia and other respiratory diseases, gynaecology, general mobility in geriatric populations

After applying these limiters in EndNote, we narrowed the references down to 343. Using Microsoft Excel, we categorized these 343 records based upon differing topics of interest. We began excluding records with the following criteria:

Duplicates

Irrelevant Record

Excluded for Language

ICU= Mobilization in the ICU (Mobilization, Early Rehabilitation, Ambulation, mechanically ventilated) or ICU topics

GM=Geriatric Mobilization (general mobilization/ambulation/movement in older adults)

CR= Cardiac rehabilitation (general mobility/ambulation/exercise after non-MI cardiac procedures

CIP=Critically Ill Patient Interventions (mobilization, ambulation, exercise)

With 117 records remaining of relevant interest, we then grouped references into 10 categories:

1. EMPMI=Early Mobilization Post MI (Mobilization, Early Rehabilitation, Ambulation)
2. HDM= Hemodynamics of Mobilization Post-MI
3. HMI=History of Early Mobilization Post-MI
4. MCD=Mobilizing with Cardiac Critical Care Devices and Therapies
5. PG=Cardiovascular Professional Guidelines
6. MIM=MI Mobilization/ambulation/Rehabilitation (not early movements)
7. ICU= Mobilization in the ICU (Mobilization, Early Rehabilitation, Ambulation, mechanically ventilated) or ICU topics
8. GM=Geriatric Mobilization (general mobilization/ambulation/movement in older adults)
9. CR= Cardiac rehabilitation (general mobility/ambulation/exercise after non-MI cardiac procedures
10. CIP=Critically Ill Patient Interventions (mobilization, ambulation, exercise)

Preferred Reporting Items for Systematic reviews and Meta-Analyses extension for Scoping Reviews (PRISMA-ScR) Checklist

SECTION	ITEM	PRISMA-ScR CHECKLIST ITEM	REPORTED ON PAGE #
**TITLE**			
Title	1	Identify the report as a scoping review.	1
**ABSTRACT**			
Structured summary	2	Provide a structured summary that includes (as applicable): background, objectives, eligibility criteria, sources of evidence, charting methods, results, and conclusions that relate to the review questions and objectives.	2
**INTRODUCTION**			
Rationale	3	Describe the rationale for the review in the context of what is already known. Explain why the review questions/objectives lend themselves to a scoping review approach.	3
Objectives	4	Provide an explicit statement of the questions and objectives being addressed with reference to their key elements (e.g., population or participants, concepts, and context) or other relevant key elements used to conceptualize the review questions and/or objectives.	4
**METHODS**			
Protocol and registration	5	Indicate whether a review protocol exists; state if and where it can be accessed (e.g., a Web address); and if available, provide registration information, including the registration number.	4
Eligibility criteria	6	Specify characteristics of the sources of evidence used as eligibility criteria (e.g., years considered, language, and publication status), and provide a rationale.	4
Information sources*	7	Describe all information sources in the search (e.g., databases with dates of coverage and contact with authors to identify additional sources), as well as the date the most recent search was executed.	5
Search	8	Present the full electronic search strategy for at least 1 database, including any limits used, such that it could be repeated.	1 of S1 Fig.
Selection of sources of evidence†	9	State the process for selecting sources of evidence (i.e., screening and eligibility) included in the scoping review.	5
Data charting process‡	10	Describe the methods of charting data from the included sources of evidence (e.g., calibrated forms or forms that have been tested by the team before their use, and whether data charting was done independently or in duplicate) and any processes for obtaining and confirming data from investigators.	5
Data items	11	List and define all variables for which data were sought and any assumptions and simplifications made.	5
Critical appraisal of individual sources of evidence§	12	If done, provide a rationale for conducting a critical appraisal of included sources of evidence; describe the methods used and how this information was used in any data synthesis (if appropriate).	N/A

SECTION	ITEM	PRISMA-ScR CHECKLIST ITEM	REPORTED ON PAGE #
Synthesis of results	13	Describe the methods of handling and summarizing the data that were charted.	5
**RESULTS**			
Selection of sources of evidence	14	Give numbers of sources of evidence screened, assessed for eligibility, and included in the review, with reasons for exclusions at each stage, ideally using a flow diagram.	5, 6
Characteristics of sources of evidence	15	For each source of evidence, present characteristics for which data were charted and provide the citations.	6-15
Critical appraisal within sources of evidence	16	If done, present data on critical appraisal of included sources of evidence (see item 12).	N/A
Results of individual sources of evidence	17	For each included source of evidence, present the relevant data that were charted that relate to the review questions and objectives.	6-15
Synthesis of results	18	Summarize and/or present the charting results as they relate to the review questions and objectives.	6-15
**DISCUSSION**			
Summary of evidence	19	Summarize the main results (including an overview of concepts, themes, and types of evidence available), link to the review questions and objectives, and consider the relevance to key groups.	15-20
Limitations	20	Discuss the limitations of the scoping review process.	20
Conclusions	21	Provide a general interpretation of the results with respect to the review questions and objectives, as well as potential implications and/or next steps.	20
**FUNDING**			
Funding	22	Describe sources of funding for the included sources of evidence, as well as sources of funding for the scoping review. Describe the role of the funders of the scoping review.	21

JBI = Joanna Briggs Institute; PRISMA-ScR = Preferred Reporting Items for Systematic reviews and Meta-Analyses extension for Scoping Reviews.
* Where *sources of evidence* (see second footnote) are compiled from, such as bibliographic databases, social media platforms, and Web sites.
† A more inclusive/heterogeneous term used to account for the different types of evidence or data sources (e.g., quantitative and/or qualitative research, expert opinion, and policy documents) that may be eligible in a scoping review as opposed to only studies. This is not to be confused with *information sources* (see first footnote).
‡ The frameworks by Arksey and O'Malley (6) and Levac and colleagues (7) and the JBI guidance (4, 5) refer to the process of data extraction in a scoping review as data charting.
§ The process of systematically examining research evidence to assess its validity, results, and relevance before using it to inform a decision. This term is used for items 12 and 19 instead of "risk of bias" (which is more applicable to systematic reviews of interventions) to include and acknowledge the various sources of evidence that may be used in a scoping review (e.g., quantitative and/or qualitative research, expert opinion, and policy document).

From: Tricco AC, Lillie E, Zarin W, O'Brien KK, Colquhoun H, Levac D, et al. PRISMA Extension for Scoping Reviews (PRISMAScR): Checklist and Explanation. Ann Intern Med. 2018;169:467–473. doi: 10.7326/M18-0850.
