# Supplementary material for: Early mobilization post-myocardial infarction: A scoping review
Source: PLoS One. 2020 Aug 17;15(8):e0237866. doi: 10.1371/journal.pone.0237866 (PMC7430744; doi:10.1371/journal.pone.0237866)
Supplement: S1 Table — (PDF) [file pone.0237866.s002.pdf]

S1 Table. Historical Evidence and Recommendations for Early Mobilization Post-MI.

Study/Year	Results	EM Recommendation
Levine, S. A. **1929**	N/A	Bedrest is most "rigid application" of ACT, when the patient is placed flat in bed for three to six weeks.
Levine, S. A. **1944**	N/A	*"It has been our view that recumbency in bed affords less rest to the heart than the sedentary position in a chair with the feet down."*
T. R. Harrison. **1944**	N/A	*"Under controlled experimental conditions excessive restriction of muscular activity was harmful, while the return to normal activity within a few days seemed to exert no detectable injurious effects."*
W. Irvin Jr.. **1950**	N/A	*"It is our considered opinion that routine prolonged bedrest in MI is not only unnecessary but also potentially harmful to the mental and physical well-being of the patients."*
Brummer, P. **1956**	Report on 322 pts from 1952-1954 indicates increased ambulation is not associated with greater hazard to pts. Was higher than normal incidence of recurrent MI after 1-month DC.	*"It is our impression that the patients have maintained their physical condition better and have regained their normal activity considerably sooner than patients under the old regimen of prolonged bedrest."*
Brummer, P. **1961**	Continued earlier ambulation therapy (mobilizing on day 12 instead of 16), with continued anticoagulant therapy (previous studies discontinued upon mobilization). Mobilized on 12th day. MIs dropped during 1st month of hospital DC from 9.3 to 2.5%, concluding that anticoagulants + mobilization rather than mobilization alone is beneficial.	*"Results from study of both series of cases clearly indicate that early ambulation is not accompanied by an increased risk to the patient with myocardial infarction."*

Miller, R.L. **1965**	N/A	*"In contrast to simple bedrest, the major loss of red cell mass was noted at the end of bedrest and not during ambulation following bedrest."*
Brummer, P. **1966**	Retrospective survey of 1682 MI patients in Finland found that the average length of bedrest was reduced from 16.2 days in 1952-54 to 10.2 days in 1962-64, with no difference in the complication-rate	N/A
Groden et al. **1966**	Found no difference in the frequency of complications of post-MI patients in a group treated with 14 days' bedrest compared with CG treated with 25 days' bedrest.	N/A
A. A. J. Adgey. **1969**	N/A	*"It is suggested that in the management of myocardial infarction the emphasis should be on early admission rather than on a prolonged period in hospital."*
J. Takkunen. **1970**	Compared mortality of two series of 254 patients post-MI divided into early (7-14 days bedrest) or late series (21 -28 days bedrest) and found no significant differences at 7- or 30-days post-MI, favoring early ambulation than generally stated in the literature	*"Results are in favour of early ambulation and suggest that the appropriate length of stay may be shorter than generally stated in the literature."*
B. M. Groden. **1970**	Two groups of male patients post-MI treated by alternative regimes of early or late mobilisation were given psychological tests on discharge and ~1yr follow up. Results show EM group had lower neuroticism scores at DC; no significant difference between early and late mobilised groups in extroversion and neuroticism scores at 1 yr FU.	*"It is suggested that the initial advantages of earlier mobilisation in producing optimism in the patient may be lost when the patient is returned to his home environment."*
R. F. DeBusk. **1971**	10 pts. post-MI participated in rehabilitation program 3-days post-MI consisting of mild, graded exercise, early sitting posture and early ambulation. No complications found in any of the patients after the program.	*"In selected patients, this (early mobilization) program is safe, simple, does not prolong hospitalization, and may contribute to earlier and more complete rehabilitation."*

Harpur. **1971**	CG (104 pts): 21 days of bedrest, mobilization day 21, discharge on day 28. Intervention (95pts): 7 days of bedrest, "EM" starting day 8, discharge on day 14. Follow-up 8 months post-DC: no difference in mortality, complication rates and ventricular aneurysm or return to work.	*"It is, therefore, advantageous from many aspects - psychological, economic, and social-to mobilise patients who have sustained an acute cardiac infarct after a shorter period of time than has generally been practised."*
M. Duke. **1971**	Evaluated bedrest physician practices in acute MI patients <65. Mean duration of bedrest ordered was 7.4 to 15.2 days (with similar patient populations). Mean duration of LOS 24 days.	*"Many patients still appear to be kept in bed and probably in the hospital for excessive and arbitrary periods of time that are not dictated by known facts"*
Shah. **1972**	111 pts post-MI. in India were not prescribed bedrest upon admission. First-four-weeks mortality was 13%, one-year mortality 21.4%, and two-year mortality 29%. Concluded that physical activity post-MI was associated with reduced mortality.	"*We concluded that physical activity after myocardial infarction was associated with reduced mortality."*
G. R. Royston. **1972**	200 consecutive males post-MI treated with minimal bedrest and early return to work. 40% DC within 2wks, 87% within a month, with no ill effects during hospital or 6-month follow up. Concluded rapid rehab is safe and desirable.	*"Rapid rehabilitation is both possible and desirable."*
J.A. Boyle. **1972**	538 pts. AMI randomly allocated to either mobilize on day 7 with DC @ day 21 or mobilize on day 21 with DC @ day 28. Found no differences between groups in mortality @ day 28, 3 months and 1 yr. Suggests EM in AMI patients may be taken 7-days post-admission.	*"Mobilisation of patients less than seventy years of age with acute but uncomplicated myocardial infarction may be safely undertaken on the seventh day after admission to hospital."*
Lamers HJ. **1973**.	203 patients post-MI mobilized either on day 10 (intervention) or day 20 (control). Patients kept in hospital for total of 30 days. Found no difference in clinical outcomes between the groups. Concluded post-MI pts. can be safely mobilized after 1-2 weeks and DC after ~3 weeks.	*"Patients with an uncomplicated myocardial infarction may safely be mobilized after 9 days and discharged after three weeks."*

AM Hutter Jr. **1973**	Prospective randomized control study comparing 2 or 3-week hospital stay in 138 pts. with uncomplicated MI. Observed no difference between patients mobilized "early" or "late" in terms of morbidity or mortality.	*“It appears that an abbreviated hospital stay for appropriately selected patients would yield substantial savings in medical-care dollars and hospital-bed utilization without diminishing the quality of clinical care for the individual patient.”*
H. H. Tucker. **1973**	342 pts. admitted to CCU with AMI assessed results EM and DC. 22.2% mortality, 7.6% of pts. readmitted. Authors conclude that results justify short hospital stay for acute MI.	*“We conclude that a shorter period in hospital and more rapid mobilization than are normally practised are justified.”*
A Bloch**. 1974**	RCT of 193 pts <age 70 with uncomplicated MI. EM is either 24 hours or 48 hours post-MI. Control is 3 weeks. Found decreased length of stay (mean of 21.3 days in intervention, vs. 32.8 in control), and no statistically significant between groups in hospital or follow-up mortality, rate of reinfarction, arrhythmias, heart failure, angina pectoris or ventricular aneurysm, or results of an exercise test. Was significantly greater disability in control that treated group on follow-up examination.	*“Early mobilization is not responsible for any of the classically described complications of myocardial infarction. It allows considerably shorter periods of hospitalization without greater risk, and it offers a wide range of advantages-physical, psychological, economic and professional.”*
Hayes MJ. **1974**	RCT of 189 pts. with uncomplicated MI. CG mobilized at 9 days, DC at 16 days. Intervention mobilized at 48 hours, discharge at 9 days. FU 6-weeks post-DC shows no difference in mortality or morbidity.	*“Clinical assessment of myocardial infarction patients at 48 hours is a reliable means of selecting the uncomplicated cases and that immediate mobilization of these patients is not associated with any increase in mortality.”*
N. C. Chaturvedi. **1974**	Prospective study of 232 pts. with AMI that were safely allowed home on the 7th day in hospital. 40% of these patients survived to the 6th day, and 68% of these patients discharged the 7th day. No deaths in these patients during 3-month FU.	*“We feel that our simple selection procedure allows a significant group of patients to be allowed home safely after only a week in hospital, and so permits a unit to concentrate its resources on patients early in the infarction stage, and for longer periods on patients at an increased risk.”*

Abraham. **1975**	Prospective RCT AMI pts... 64 pts. mobilized on day 6, DC day 12. 65 patients mobilized on day 13, DC 19. FU showed early ambulation is beneficial irrespective of complications	*"We conclude that early ambulation is beneficial irrespective of complications on admission."*
Swan HJ. **1976**	Ad hoc review of clinical and laboratory findings of acute MI pts.. Recommend that EM program with progressive activity over 5 to 10 days should reduce LOS to less than current average of 17.5 to 20.8 days for AMI pts.	*"If by the 5th hospital day no complication is evident, mobilization by the 7th to 10th day and discharge from the hospital by approximately the 14th day is not associated with increased risk."*
Jelinek, V. **1977**	Defined low-risk after MI. 30/189 pts. met these criteria. Gave early exercise testing 1-5 weeks post- admission; conclude early exercise provide useful guidelines for returning to work and recommend it as integral part in rehab.	*"Early exercise testing proved useful in providing guidelines for return to most work, leisure, and sexual activities within four weeks of admission to hospital… we recommend it as an integral part in the rehabilitation of these patients."*
McNeer JF. **1978**	67 pts. with acute-MI in trial for DC within 1 week. 33/67 pts. discharged within 1 week. Found no serious complications in either groups at 3-weeks FU and no deaths or difference in functional status at 6-months FU in either groups.	*"It is feasible and ethically justified to discharge such uncomplicated patients at one week after an acute myocardial infarction. The potential economic savings through earlier discharge in these patients are of major importance."*
Beamish, R. **1977**	32 patients post-MI remained ambulant and observed for 6 months. After 6 months, all but 2 were well before their MI. Suggest moderate activity for pts. to avoid "undesirable consequences" of bedrest	*"Our experience suggests that selected patients can be allowed moderate activity without ill effects and thus avoid the undesirable consequences of enforced bedrest."*
Thornley, P. **1977**	142 men <65 years of age after acute-MI in the CCU divided into 3 mobilization times: 1. 2-4 days in bed (n=74), 2. 5-10 days in bed (n=42) or 3. >10 days in bed (n=11). Mean bed-rest period was 5.4 days. Rapid mobilization led to earlier DC.	*"It is clear that rapid mobilization and early discharge after myocardial infarction should now be standard practice and there is no need of further evidence of its safety."*
Lindvall. **1979**	n=184 pts in the CCU (48 hrs). 2 groups: 1) Rapidly mobilized (RM) (n=55) 2) CG (n=129). 42 RM patients mobilized and discharged in a mean stay of 9 days in contrast to mean of 19 days	*"Early exercise test in selected good risk patients is safe and identifies a group prone to complications during the early follow-up period."*

	in the CG group. Early exercise test in selected patients is safe in AMI pts.	
West, R. **1979**	n=742 pts in 13 hospitals randomly allocated to EM either on 5th or 10th day after MI. Found no difference in 1st year mortality or morbidity between groups	*"Early mobilization in practice thus led to earlier discharge and resulted in lower costs"*
Kohn, R. M.. **1982**	N/A	*"It seems strange that in the 1980s we continue to debate the dangers of early ambulation for the patient with myocardial infarction."*
Wenger, N. K. **1982**	Questionnaires sent to 6000 physicians to determine pattern in changes of care between 1970-1980 for pts. with uncomplicated AMI. Found early ambulation and return to work are more common practices	N/A
Magder, S. **1985**	Measured HR, BP, and rhythm in n=32 pts during sitting, standing and walking within first 2 days post-MI. Ambulatory activities caused small changes in HR, BP unchanged or decreased. Concluded that mild ambulatory activities within first few days of MI can be permitted, as long as BP monitored.	*"Mild ambulatory activities produce little stress for the myocardium and can be permitted in the first few days following infarction as long as blood pressure is measured."*

ACT, Acute coronary thrombosis; AMI, Acute myocardial infarction; BP, Blood pressure; CCU, Cardiac care unit; CG, Control group; DC, discharge; EM, Early mobilization; FU, Follow-up; HR, Heart rate; IG=Interventional group; LOS= Length of stay; MI, Myocardial infarction; Pts, Patients; RCT, Randomized control trial.
